# ABT-263 Reduces Hypertrophic Scars by Targeting Apoptosis of Myofibroblasts

**DOI:** 10.3389/fphar.2020.615505

**Published:** 2021-01-14

**Authors:** Xiaolan Yang, Yongqiang Xiao, Chenjian Zhong, Futing Shu, Shichu Xiao, Yongjun Zheng, Zhaofan Xia

**Affiliations:** ^1^ Department of Burn Surgery, Changhai Hospital, Naval Medical University, Shanghai, China; ^2^ Fujian Burn Institute, Fujian Medical University Union Hospital, Fuzhou, China; ^3^ Research Unit of Key Techniques for Treatment of Burns and Combined Burns and Trauma Injury, Chinese Academy of Medical Sciences, Shanghai, China; ^4^ Quanzhou First Hospital Affiliated to Fujian Medical University, Fujian, China; ^5^ Department of Burn and Plastic Surgery, The 970th Hospital of People’s Liberation Army, Yantai, China

**Keywords:** ABT-263, hypertrophic scar, apoptosis, myofibroblast, skin

## Abstract

**Background:** Inhibiting proliferation and inducing apoptosis of myofibroblasts is becoming one of the promising and effective ways to treat hypertrophic scar. ABT-263, as an orally bioavailable BCL-2 family inhibitor, has showed great antitumor characteristics by targeting tumor cell apoptosis. The objective of this study was to explore whether ABT-263 could target apoptosis of overactivated myofibroblasts in hypertrophic scar.

**Methods:** *In vivo*, we used ABT-263 to treat scars in a rabbit ear scar model. Photographs and ultrasound examination were taken weekly, and scars were harvested on day 42 for further Masson trichrome staining. *In vitro*, the expression levels of BCL-2 family members, including prosurvival proteins, activators, and effectors, were detected systematically in hypertrophic scar tissues and adjacent normal skin tissues, as well as in human hypertrophic scar fibroblasts (HSFs) and human normal dermal fibroblasts (HFBs). The roles of ABT-263 in apoptosis and proliferation of HSFs and HFBs were determined by annexin V/PI assay, CCK-8 kit, and cell cycle analysis. Mitochondrial membrane potential was evaluated by JC-1 staining and the expression of type I/III collagen and α-SMA was measured by PCR, western blotting, and immunofluorescence staining. Furthermore, immunoprecipitation was performed to explore the potential mechanism.

**Results:** *In vivo*, ABT-263 could significantly improve the scar appearance and collagen arrangement, decrease scar elevation index (SEI), and induce cell apoptosis. *In vitro*, the expression levels of BCL-2, BCL-XL, and BIM were significantly higher in scar tissues and HSFs than those in normal skin tissues and HFBs. ABT-263 selectively induced HSFs apoptosis by releasing BIM from binding with prosurvival proteins. Moreover, ABT-263 inhibited HSFs proliferation and reduced the expression of α-SMA and type I/III collagen in a concentration- and time- dependent manner.

**Conclusion:** HSFs showed increased mitochondrial priming with higher level of proapoptotic activator BIM and were primed to death. ABT-263 showed great therapeutic ability in the treatment of hypertrophic scar by targeting HSFs.

## Introduction

Hypertrophic scar affects millions of patients, particularly children and burn victims, resulting in long-term physical dysfunction and psychological stress (Sheridan et al., 2000; Cen et al., 2015). Currently, there are a variety of treatments for hypertrophic scars, such as resection, corticosteroid injection, compression, and laser treatment. However, there remains unsatisfactory treatment to permanently eliminate scars (Bhargava et al., 2018; Lv and Xia, 2018). Therefore, it is urgently needed to find more effective and satisfactory treatments.

Although the exact pathogenesis of hypertrophic scar is not fully elucidated, myofibroblasts play a pivotal role in scar formation, characterized by increased expression of α-smooth muscle actin (α-SMA), excessive collagen synthesis, immoderate cell proliferation, and reduced cell apoptosis (Hinz, 2016). Myofibroblasts are responsible for tissue contraction and excessive extracellular matrix (ECM) secretion during hypertrophic scar formation, leading to stiffness, compression, and loss of joint mobility. Strategies that inhibit myofibroblast proliferation and promote myofibroblast apoptosis are effective ways for treating hypertrophic scar (Shi et al., 2018; Zhou et al., 2018).

ABT-263 is a small-molecule BH3 mimetic drug and inhibitor of prosurvival BCL-2 family proteins, which could directly bind to and block prosurvival BCL-2 family members (BCL-2 and BCL-W and BCL-XL) and then activate intrinsic apoptosis pathway (Wilson et al., 2010; Vogler et al., 2011). Recently, ABT-263 has been widely used as an orally bioavailable inhibitor of prosurvival BCL-2 proteins in many antitumor therapies by targeting tumor cell apoptosis and inhibiting cell proliferation (Gandhi et al., 2011; Wang et al., 2016; Froehlich et al., 2019). However, whether it can reduce hypertrophic scars remains unclear. In this study, we explored the therapeutic effects of ABT-263 on hypertrophic scar *in vivo* and further explored related mechanisms by *in vitro* experiments, with a focus on the direct effects of ABT-263 on targeting myofibroblasts apoptosis.

## Materials and Methods

### Reagents

Antibodies used were listed as follows: anti-collagen I antibody, anti-collagen III antibody (Gene Tex, USA); anti-BIM, BCL-2, BCL-XL, and α-SMA antibody (Abcam, USA); anti-tubulin antibody (Cell Signaling Technology, USA). Secondary antibodies Alexa Fluor 488 goat anti-mouse immunoglobulin IgG2a and Alexa Fluor 555 goat anti-rabbit IgG1 were purchased from Thermo Fisher Scientific (Waltham, MA, USA). ABT-263 was purchased from Selleck Chemicals (Houston, TX, USA), dissolved in DMSO (Beyotime, Shanghai, China). Cell cycle staining kit and annexin V apoptosis kit were obtained from BD Biosciences (San Jose, CA). Cell counting kit-8 kit and mitochondrial membrane potential assay kit with JC-1 were purchased from Beyotime (Shanghai, China), and coimmunoprecipitation kit was purchased from Thermo Fisher Scientific (Waltham, MA, USA).

### Rabbit Ear Hypertrophic Scar Model Establishment and Treatment

All animal studies were approved by the Ethics Committee of Changhai Hospital, Shanghai, China (NO. CHEC2014-096). As described in our previous study, a hypertrophic scar model was established by using rabbit ears (Xiao et al., 2019). Rabbits were housed in individual cages in controlled conditions (23 ± 3 °C, 50 ± 10% humidity, and 12 h day/night cycle) with free access to food and water. Eight male New Zealand rabbits aged 3 months and weighting 2.5–3 kg were anesthetized with pentobarbital sodium (1mg/kg). Six circular full-thickness wounds with diameter 10 mm were made on the ventral surface of each ear by removing the epidermis, dermis, and perichondrium to the bare cartilage. Animals received systemic analgesia after surgery through remifentanil being administered at a dose of 10 μg kg^−1^ h^−1^. The rabbits were randomly divided into the control group and the experimental group. On postoperative day 14, the wounds were completely reepithelialized and then injected with ABT-263 dissolved in DMSO (150μM, 100 μL) in the experimental group, while the wounds in the control group were injected with 100 μL DMSO only. ABT-263 or DMSO was carefully injected into the center of each lesion from the edge of the wound with a 29-G needle. The injections were administered once a week for a total of 4 times. The dose of ABT-263 was measured in preliminary experiments where wounds were given 50, 150, 250, and 350 μM in 100 μL DMSO. The 150 μM ABT-263 in 100 μL DMSO was the minimum required to achieve the greatest attenuation of scar elevation index. In addition, drug administration was preliminarily performed with different time intervals, namely, once a day, once every three days, and once every week. Once every week was the minimum required to avoid skin necrosis.

### Scar Evaluation and Histologic Analysis

The gross appearance of each wound was monitored every week by two scar specialists, and the thickness of the scar was calculated by ultrasound (MyLabOne, Italy). SEI (scar elevation index) provided an accurate parameter to assess scar formation and was calculated as follows: SEI = H/H_0_, where H represents the length between the highest point to the surfaces of cartilage in scar, and H_0_ represents the length from the epithelium to the surface of the cartilage in adjacent normal skin (Zhang et al., 2015; Lin et al., 2019; Feng et al., 2020).

The specimen was collected on day 42 after wounding, divided equally in half, one half of the specimen for histologic analysis and the other half for western blot. For histologic analysis, the specimen was fixed in 4% formaldehyde solution for 24 h, embedded in paraffin, cut into 5 mm slices, and then analyzed by Masson trichrome staining.

### Terminal Deoxynucleotidyl Transferase-Mediated dUTP Nick End Labeling Staining

TUNEL staining was performed using an apoptosis detection kit (Roche, California, USA) following the manufacturer’s instructions. Briefly, rabbit ear sections were deparaffinized, hydrated, and permeabilized with 0.1% Triton X-100. After being washed twice with PBS, the sections were stained in sequence with TUNEL test solution and DAPI. The images were then taken under the fluorescence microscope (Olympus, Tokyo, Japan).

### Human Normal Dermal Fibroblasts and Hypertrophic Scar Fibroblasts Isolation and Culture

All experimental procedures have been approved by the Ethics Committee of Changhai Hospital, Shanghai, China. After obtaining informed consent, we collected the tissues of hypertrophic scar from six patients (three males, three females) who underwent surgical excision of hypertrophic scar in the Changhai Hospital. After being incubated with type I collagenase (0.1 mg/ml, Sigma) at 37 °C for 4 h, HSFs and HFBs were isolated from human hypertrophic scar tissue and adjacent normal skin tissue, respectively. The extracted cells were cultured in Dulbecco's Modified Eagle Media (DMEM) supplemented with 10% FBS, 1% penicillin, and streptomycin. Cells between passage two and passage four were used for further experiments. HSFs are α-SMA positive, while HFBs are α-SMA negative, as measured by immunofluorescence staining.

### Quantitative Real-Time PCR

HSFs and HFBs were cultured on 6-well culture plates and treated with or without ABT-263 (0, 5, 15, and 25 μM) for 24 h. The total RNA of cells and skin tissues was isolated using Trizol reagent (Life Technologies, USA) and then quantitative real-time PCR was performed using SYBR Green PCR Master Mix in a total volume of 10 ml with the Step One Plus Real-Time PCR System (Applied Biosystems) as previously reported method (Zheng et al., 2017). GAPDH was served as an internal reference and primer sequences used were listed as follows:

BCL-2, 5′-GGT​GGG​GTC​ATG​TGT​GTG​G-3′ and 5′-CGG​TTC​AGG​TAC​TCA​GTC​ATC​C-3′; BCL-XL, 5′-GAG​CTG​GTG​GTT​GAC​TTT​CTC-3′ and 5′-TCC​ATC​TCC​GAT​TCA​GTC​CCT-3′; BCL-W, 5′-GCG​GAG​TTC​ACA​GCT​CTA​TAC-3′ and 5′-AAA​AGG​CCC​CTA​CAG​TTA​CCA-3′; BIM, 5′-TAA​GTT​CTG​AGT​GTG​ACC​GAG​A-3′ and 5′-GCT​CTG​TCT​GTA​GGG​AGG​TAG​G-3′; MCL-1, 5′-TGC​TTC​GGA​AAC​TGG​ACA​TCA-3′ and 5′-TAG​CCA​CAA​AGG​CAC​CAA​AAG-3′; BAX, 5′-CCC​GAG​AGG​TCT​TTT​TCC​GAG-3′ and 5′-CCA​GCC​CAT​GAT​GGT​TCT​GAT-3′; BAK, 5′-GTT​TTC​CGC​AGC​TAC​GTT​TTT-3′ and 5′-GCA​GAG​GTA​AGG​TGA​CCA​TCT​C-3′; α-SMA,5′-AGGTAACGAGTCAGAGCTTTGGC-3′ and 5′-CTCTCTGTCCACCTTCCAGCAG-3; collagen 1, 5′-GAGGGCCAAGACGAAGACATC-3′and 5′-CAGATCACGTCATCGCACAAC-3′; collagen 3, 5′-GGA​GAC​GGC​TAT​TTT​GGG​ACG-3′ and 5′-TCC​TTG​AGT​GGA​GCT​TCC​ATT-3′; GAPDH, 5′-AGA​ACA​TCA​TCC​CTG​CAT​CC-3′ and 5′-TCC​ACC​ACC​CTG​TTG​CTG​TA-3′.

### Annexin V/PI Staining

HSFs and HFBs were cultured on 6-well culture plates and treated with or without ABT-263 (0, 5, 15, and 25 μM) for 24 h or 48 h. Apoptotic cells were measured by using an annexin V apoptosis detection kit following the manufacturer’s instructions. In brief, 100 μL cell suspension was incubated with 5 μL FITC-annexin V for 15 min and then incubated with 5 μL propidium iodide (PI) staining solution for another 10 min at room temperature. Finally, the percentage of apoptotic cells was detected using flow cytometer (Beckman, Los Angeles, USA).

### Cell Cycle Assay

HSFs were cultured with different concentrations of ABT-263 (0, 5, 15, and 25 μM) for 24 h. The cells were collected using 0.25% trypsin, washed twice with prechilled phosphate-buffered saline (PBS), and added with 1 ml DNA staining solution and 10 μL permeation solution according to the manufacturer’s instructions. After being incubated in the dark for 30 min, the samples were analyzed through a flow cytometer (Beckman, Los Angeles, USA).

### Mitochondrial Membrane Potential

HSFs and HFBs were cultured on 6-well culture plates with or without ABT-263 (0, 5, 15, and 25 μM) for 48 h. Following the manufacturer’s instructions of JC-1 assay kit, the cells were washed with prechilled PBS, added with 1 ml JC-1 staining working solution to each well, and incubated for 30 min. The mitochondrial membrane potential changes were observed through a fluorescent microscope. Red fluorescence represented JC-1 aggregate in mitochondria and green fluorescence indicated JC-1 monomer in mitochondria.

### Western Blotting

Cell lysates were obtained from the skin samples and treated cells with lysis buffer containing proteinase inhibitors. After being centrifuged at 12,000 rpm for 20 min at 4 °C, the supernatants were harvested for further western blot analysis. The protein concentration was measured by a Pierce BCA Protein Assay Kit (Thermo Fisher Scientific, USA), and then immunoblotting was performed with corresponding primary antibodies and secondary antibodies.

### Coimmunoprecipitation

Coimmunoprecipitation was performed by Pierce Co-Immunoprecipitation Kit (Thermo Fisher Scientific, USA) according to the manufacturer’s instructions. Briefly, 25 μg antibody was fixed to AminoLink Plus Coupling Resin and washed three times before the antibody-conjugated resin was ready for IP. Cell lysate was added to the resin and rotated for 1 h at room temperature. The precipitated proteins were eluted with elution buffer and the eluent was used for western blot analysis.

### Immunofluorescence Staining

Immunofluorescence staining was performed to analyze the expression of collagen and α-SMA in HSFs. Briefly, the cells were fixed with 4% paraformaldehyde for 30 min at room temperature, permeabilized with 0.1% Triton X-100, and then incubated with corresponding primary antibodies and fluorescent secondary antibodies. Finally, the images were taken by the fluorescence microscope (Olympus, Tokyo, Japan).

### Statistical Analysis

SPSS 22.0 software (IBM Corporation, Armonk, NY, USA) and GraphPad Prism 8.0 (La Jolla, CA, USA) were used for statistical analysis and graphical presentations. The data were presented as mean ± standard deviation (SD). Differences between two groups were analyzed with Student’s t test and differences among three groups were analyzed with one-way analysis of variance (ANOVA). *p* < 0.05 was considered as statistical significance.

## Results

### ABT-263 Reduced Scar Formation in Rabbit Hypertrophic Scar Model

**FIGURE 1:**
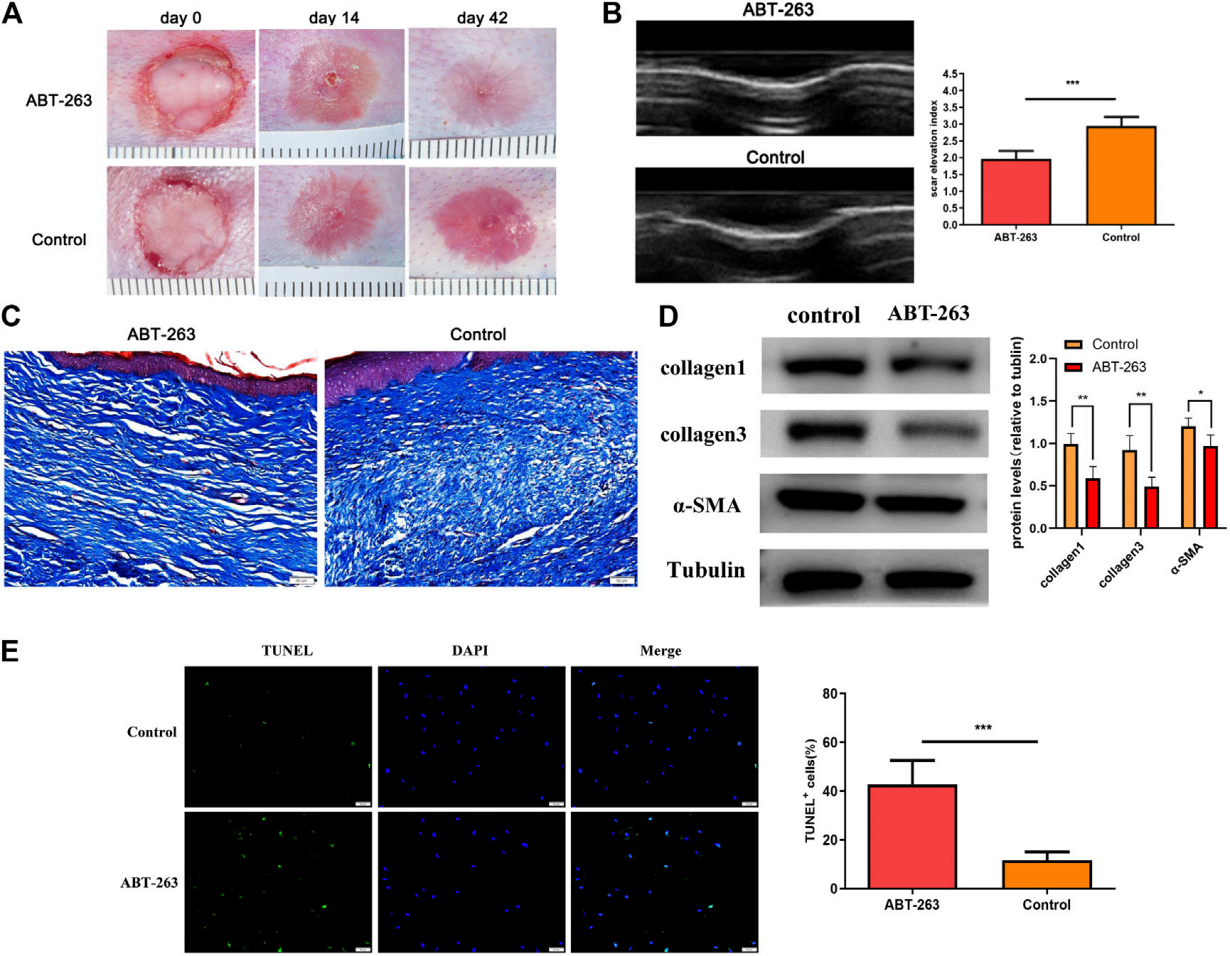
The effect of ABT-263 on hypertrophic scar *in vivo*. **(A)** Gross examination of scars after treatment with ABT-263. **(B)** The thickness of the scar on day 42 after wounding was measured by ultrasound examination and SEI (scar elevation index) was calculated. n = 5. **(C)** Masson trichrome staining of scars from different groups. Scale bars: 50 μm. **(D)** The protein expression of collagen I, collagen III, and α-SMA in day 42 wounds, measured by western blotting. n = 5. **(E)** TUNEL staining and quantification of the percentage of TUNEL-positive cells in day 42 wounds. n = 5. Scale bars: 50 μm. Data represented the means ± SD. ***p < 0.001 vs. control.

The rabbit ear hypertrophic scar model was established to determine the therapeutic role of ABT-263 *in vivo*. On day 14 after surgery, all wounds showed complete reepithelialization. On day 42 after wounding, scars were formed with stiff, red, and visible raised appearance in all wounds of control group. Nevertheless, the scars treated with ABT-263 reduced significantly, with softer and less visible appearance (Figure 1A). Consistent with gross appearance, further ultrasound examination showed that the scars treated with ABT-263 were thinner and SEI was lower than that in the control group (Figure 1B). The collagen deposition after treatment with ABT-263 was observed by Masson trichrome staining, showing significantly less and well-arranged collagen deposition in ABT-263 treated group (Figure 1C). Furthermore, the protein expression levels of collagen I, collagen III, and α-SMA were significantly lower in the ABT-263 treated group than that in the control group (Figure 1D). To evaluate the effect of ABT-263 on cell apoptosis in scar tissue, we performed TUNEL staining on rabbit ear-tissue sections. The results showed that the percentage of TUNEL-positive cells was significantly higher in the ABT-263 treated group than that in the control group (Figure 1E).

### ABT-263 Selectively Induced Human Hypertrophic Scar Fibroblasts Apoptosis *in vitro*

**FIGURE 2:**
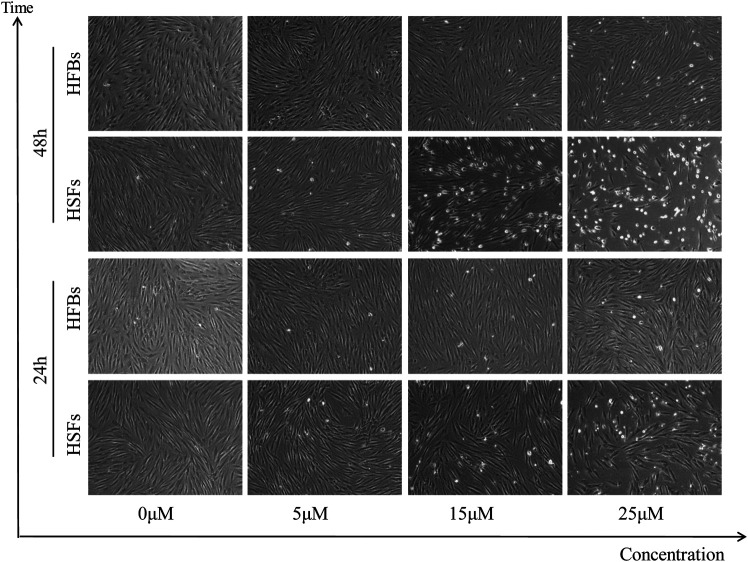
Morphological changes in HSFs and HFBs after treatment with different concentration of ABT-263. The results showed that HSFs were susceptible to ABT-263 induced apoptosis, with more floated spherical cells, while HFBs were insensitive.

**FIGURE 3:**
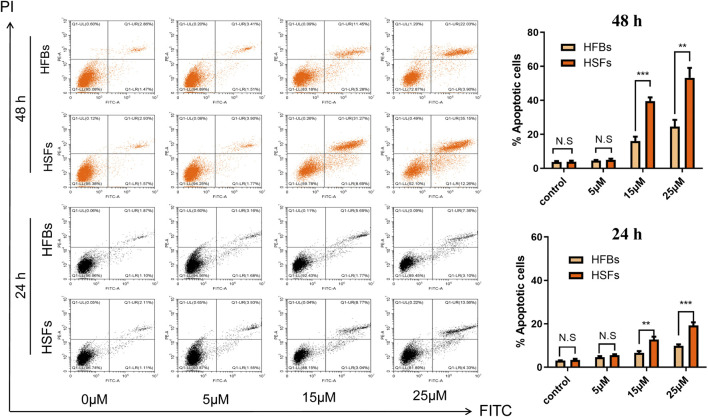
ABT-263 selectively induced apoptosis of HSFs. After double staining with annexin V-FITC and PI, the cells were quantitatively analyzed with flow cytometry, demonstrating that ABT-263 could induce more apoptosis to HSFs in a time and dose dependent manner, whereas HFBs were less sensitive for ABT-263 induced apoptosis. n = 3 and data represented the means ± SD. **p < 0.01, ***p < 0.001, and n.s., no significance.

**FIGURE 4:**
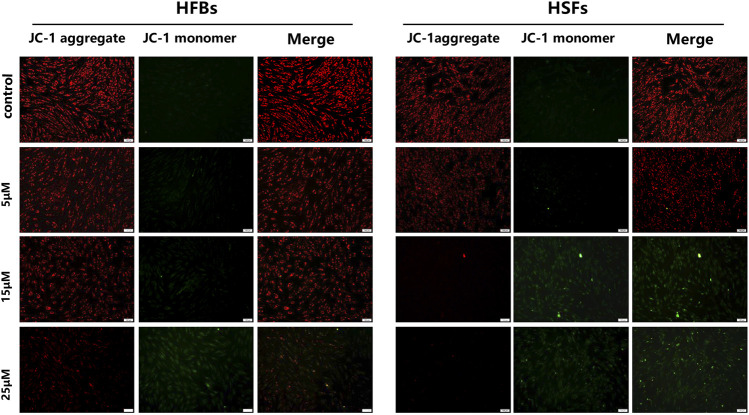
Mitochondrial membrane potential was measured by JC-1 assay kit. Red fluorescence represented JC-1 aggregate in mitochondria and green fluorescence indicated JC-1 monomer in mitochondria. The results showed that the mitochondrial membrane potential changed more obviously with the increase of ABT-263 concentration in HSFs when compared with HFBs. Scale bars: 100 μm.

To determine the *in vitro* effects of ABT-263 on human hypertrophic scar fibroblasts and human normal skin fibroblasts, HFBs and HSFs were cultured and treated with different concentrations of ABT-263 for 24 and 48 h. The gross examination showed that proliferation of HSFs was significantly inhibited by ABT-263 in both time and dose dependent manner. Most of HSFs floated spherically at 48h, while HFBs were spindle-shaped and fast-growing (Figure 2). Then annexin V and PI based flow cytometry analysis was used to measure the apoptosis in HSFs and HFBs. As shown in Figure 3, ABT-263 could induce more apoptosis in HSFs in a time and dose dependent manner, whereas HFBs were less sensitive for ABT-263 induced apoptosis. To determine whether ABT-263 induced HSFs apoptosis via mitochondria-associated pathways, HSFs and HFBs were treated by different concentrations of ABT-263 for 48 h and then JC-1 was used to detect the mitochondrial membrane potential, showing that the mitochondrial membrane potential changed more obviously with the increase of ABT-263 concentration in HSFs when compared with HFBs (Figure 4). These data indicated that ABT-263 could selectively induce HSFs apoptosis through mitochondria-associated pathways, while it caused less damage to normal skin fibroblasts.

### ABT-263 Targeted Human Hypertrophic Scar Fibroblasts Apoptosis by Releasing BIM From Binding With Prosurvival Proteins

**FIGURE 5:**
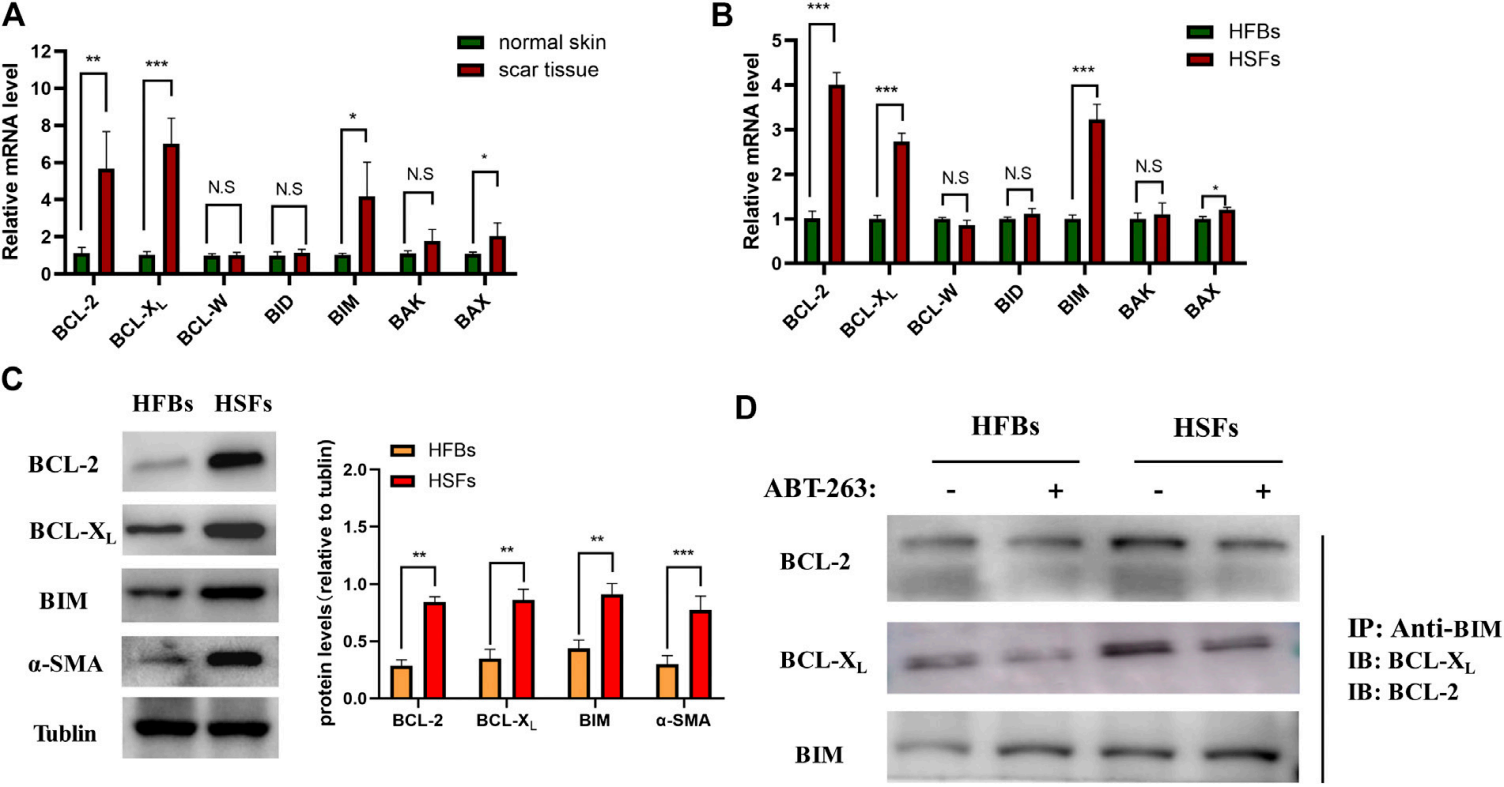
ABT-263 selectively induced HSFs apoptosis by releasing BIM from binding with prosurvival proteins. **(A)** The mRNA expression levels of BCL-2 family members in hypertrophic scar tissues and adjacent normal skin tissues, including prosurvival proteins (BCL-2, BCL-XL, and BCL-W), activators (BID and BIM), and effectors (BAX and BAK). n = 4. **(B)** The mRNA expression levels of BCL-2 family members in HFBs and HSFs, including BCL-2, BCL-XL, BCL-W, BID, BIM, BAX, and BAK. n = 3. **(C)** The protein expression levels of BCL-2, BCL-XL, and BIM in HFBs and HSFs were measured by western blotting. n = 3. **(D)** BIM is sequestered by BCL-XL and BCL-2 in HSFs and displaced by ABT-263. Anti-BIM immunoprecipitation of whole-cell lysates from HFBs and HSFs treated with or without ABT-263 for 8 h. BCL-2 and BCL-XL were immunoblotted. Data represented the means ± SD. *p < 0.05, **p < 0.01, ***p < 0.001, and n.s., no significance.

The pathway of apoptosis is tightly regulated by the BCL-2 family members, including prosurvival proteins (BCL-2, BCL-XL, and BCL-W), activators (BID and BIM), and effectors (BAX and BAK). Previous studies have demonstrated that the potential mechanism of ABT-263 proapoptotic activity depends on the release of BIM from binding with BCL-XL and BCL-2 (Tse et al., 2008). To further clarify the mechanism of ABT-263 targeting HSFs apoptosis, we first detected the expression levels of BCL-2 family members systematically. As shown in Figure 5A, the mRNA expression levels of BCL-2, BCL-XL, BAX, and BIM were significantly higher in hypertrophic scar tissues than those in adjacent normal skin tissues, whereas there was no significant difference of the mRNA expression levels of BCL-W, BID, and BAK between scar tissues and adjacent normal skin tissues. The mRNA expression levels of BCL-2 family members were also detected in HSFs and HFBs, showing similar trend as that in scar tissues and normal skin tissues (Figure 5B). The results were confirmed by western blotting, showing higher protein expression levels of BCL-2, BCL-XL, and BIM in HSFs (Figure 5C). We further performed coimmunoprecipitation of BIM/BCL-XL and BIM/BCL-2 complexes from cell lysates extracted from HSFs and HFBs after treatment with or without ABT-263. As shown in Figure 5D, the results demonstrated that ABT-263 could significantly reduce the binding of BIM to BCL-XL and BCL-2 in HSFs, which was consistent with the view that ABT-263 could induce HSFs apoptosis by releasing proapoptotic protein BIM from prosurvival proteins BCL-XL and BCL-2. As described above, mitochondria in HSFs, but not fibroblasts of normal skin tissue, are primed by death signals. This may explain why HSFs become more susceptible to apoptosis induced by BH3 mimetic drug ABT-263.

### ABT-263 Inhibited Human Hypertrophic Scar Fibroblasts Proliferation and Blocked Cell Cycle at G0/G1 Phase in a Concentration Dependent Manner

**FIGURE 6:**
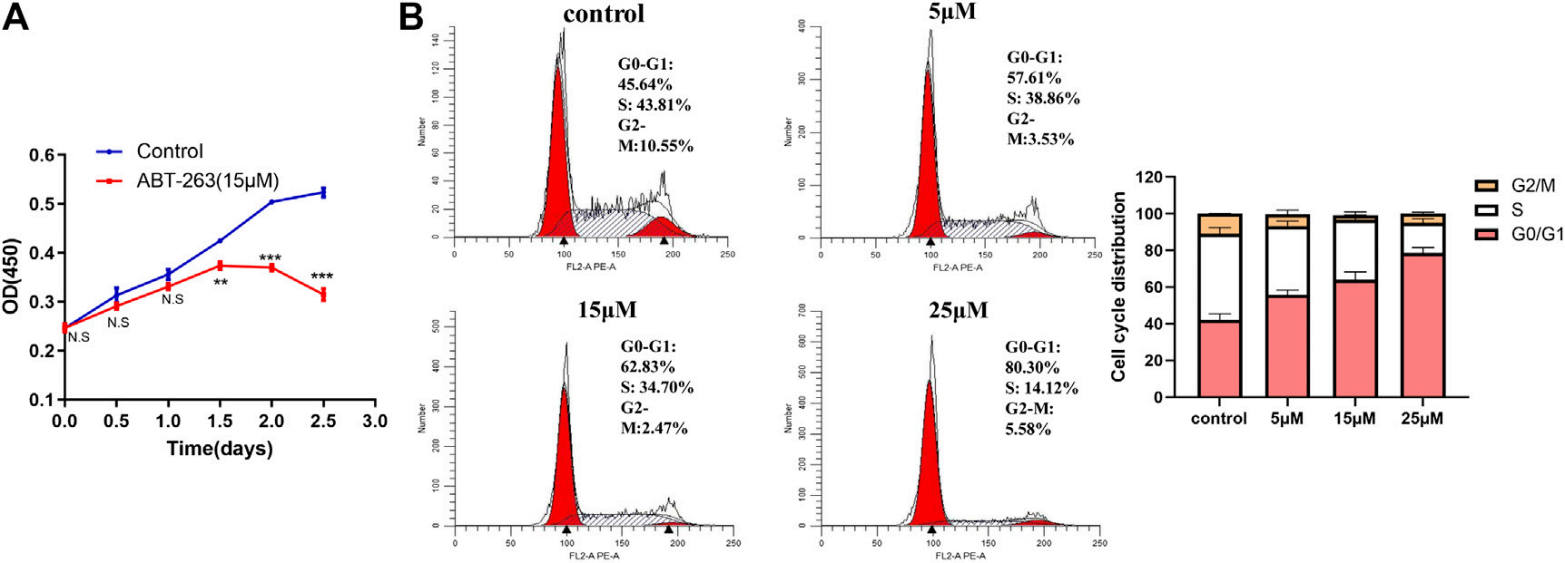
ABT-263 inhibited HSFs proliferation and blocked cell cycle. **(A)** Cell proliferation was measured by CCK-8 kit in HSFs after treatment with different concentration of ABT-263. **(B)** Cell cycle of HSFs was detected with flow cytometry and quantitative analysis was further performed. n = 3. Data represented the means ± SD.

CCK-8 assay was used to detect cell proliferation after treatment with ABT-263. The results showed that ABT-263 could inhibit the growth of HSFs significantly (Figure 6A). Next, we tested the effect of ABT-263 on the cell cycle of HSFs by flow cytometry. As shown in Figure 6B, as the concentration of ABT-263 increased, the percentage of cells in the G0/G1 phase dramatically increased, while the percentage of cells in the S phase markedly decreased and cells in the G2/M phase did not change significantly. These data indicated that ABT-263 could inhibit HSFs proliferation and block cell cycle at G0/G1 phase.

### ABT-263 Reduced the Expression of α-SMA, Collagen I, and Collagen III in Human Hypertrophic Scar Fibroblasts

**FIGURE 7:**
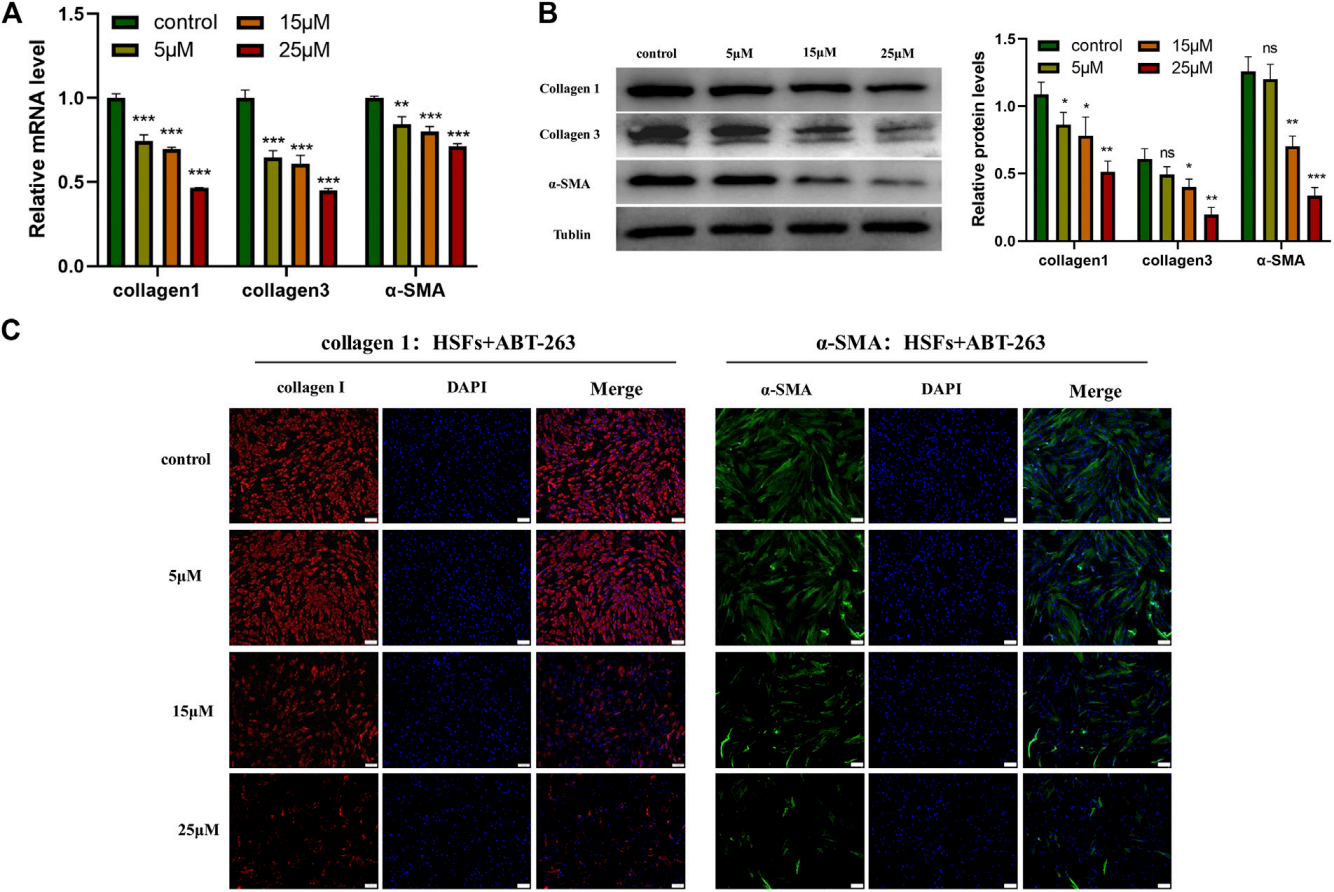
ABT-263 reduced the expression of α -SMA, collagen I, and collagen III in HSFs. **(A)** The mRNA expression levels of α -SMA, collagen I, and collagen III in HSFs after treatment with different concentration of ABT-263 were determined by PCR. n = 3. **(B)** The protein expression levels of α-SMA, collagen I, and collagen III in ABT 263-treated HSFs were determined by western blot. n = 3. **(C)** The protein expression levels of collagen I and α-SMA in ABT-263 treated HSFs were further confirmed by immunofluorescence staining. Collagen I was stained with red, α-SMA with green, and nuclei with blue. Scale bars: 100 μm. Data represented the means ± SD. *p < 0.05, **p < 0.01, ***p < 0.001, and n.s., no significance. vs. control.

To further explore the effects of ABT-263 on collagen synthesis and α -SMA expression, HSFs were treated with different concentrations of ABT-263 for 24 h. The expression of α-SMA, collagen I, and collagen III was detected by q-PCR and western blot. As shown in Figures 7A,B, the results demonstrated that ABT-263 reduced the expression of α-SMA, collagen I, and collagen III in both mRNA and protein level after treatment with different concentration of ABT-263. These results were further confirmed by immunofluorescence, showing that ABT-263 could inhibit the expression of collagen I and α-SMA in ABT-263 treated HSFs (Figure 7C). Collectively, these data suggested that ABT-263 might have the potential to treat scars by selectively inducing HSFs apoptosis, inhibiting HSFs proliferation, and reducing collagen deposition and α-SMA expression.

## Discussion

In this study, we found that the expression levels of BCL-2, BCL-XL, and BIM were significantly increased in HSFs. Based on this, we further showed that ABT-263, a targeted high-affinity inhibitor of BCL-2 family, could selectively induce myofibroblast apoptosis and inhibit hypertrophic scar formation, which indicates the potential of ABT-263 as an effective treatment for hypertrophic scar. To our knowledge, this is the first study to measure the expression of BCL-2 family members systematically and explore the role of ABT-263 in hypertrophic scar.

Evasion of apoptosis by myofibroblasts is one of the underlying mechanisms of various types of fibrotic disease (Teofoli et al., 1999; Thannickal and Horowitz, 2006; Iqubal et al., 2020; Xu et al., 2020). Apoptosis is a physiological process in which cells are genetically programmed to self-destruct, including the intrinsic and extrinsic pathways. The intrinsic pathway, called mitochondrial apoptosis, is tightly controlled by the BCL-2 family proteins, including prosurvival proteins, activators, and effectors (Youle and Strasser, 2008). Prosurvival proteins such as BCL-2 and BCL- XL could bind and sequester both activators and effectors to inhibit cell apoptosis. Once activator proteins such as BIM are released from prosurvival proteins, they activate effector protein BAX and induce apoptosis by promoting mitochondrial outer membrane permeabilization. Relative expression of proapoptotic and antiapoptotic BCL-2 family members determines the fate of cells. In the past, the persistence of myofibroblasts in fibrotic tissue has suggested that these cells are apoptosis-resistant, characterized by increased BCL-2/BAX ratio (Thannickal and Horowitz, 2006). Recently, studies have indicated that, rather than resisting apoptosis in fibrotic conditions, myofibroblasts virtually tend to be self-destructive, manifested as an increase in mitochondrial apoptotic priming (Lagares et al., 2017; Kuehl and Lagares, 2018). Mitochondrial priming indicates the closeness of mitochondria to cell apoptosis threshold and is determined by the relative expression of prosurvival proteins, effectors, and activators from the BCL-2 family (Del Gaizo Moore et al., 2007; Ryan et al., 2010). Proapoptotic effectors and activators increased mitochondrial priming and made myofibroblasts primed to death (Lagares et al., 2017). However, these cells could still survive even with a heightened mitochondrial priming when the prosurvival mechanism is activated, by upregulation of antiapoptotic BCL-2 family members, such as BCL-2 and BCL-XL (Lagares et al., 2017). Though few studies have detected the expression of BCL-2 family members in hypertrophic scar before, they only measure the expression of BCL-2 or BAX or BCL-2/BAX ratio, which cannot comprehensively show the prosurvival or proapoptotic status of hypertrophic scar (Teofoli et al., 1999; Zhang et al., 2017). In this study, we separated myofibroblasts from human hypertrophic scar and detected the expression of BCL-2 family members systematically, including prosurvival proteins (BCL-2, BCL-XL, and BCL-W), activators (BID and BIM), and effectors (BAX and BAK). In mRNA and protein levels, we found that BCL-2, BCL-XL, and BIM were significantly higher in HSFs than those in HFBs, whereas there was no significant difference of the expression of BCL-W, BID, and BAK between HSFs and HFBs. To our knowledge, this is the first time to measure the expression of BCL-2 family members in HSFs systematically, which demonstrated increased mitochondrial priming in HSFs.

ABT-263, as a potent BH3 mimetic, possesses high binding affinity to BCL-2 antiapoptotic family proteins and blocks its prosurvival function and then releases BIM, which directly activates BAX and BAK to induce cell apoptosis (Tse et al., 2008). ABT-263 has been reported to target apoptosis of primed cancer cells in various kinds of tumor based on the increased mitochondrial priming in these cancer cells (Ni Chonghaile et al., 2011; Vo et al., 2012; Czabotar et al., 2014). Here, we demonstrated that elevated mitochondrial priming of myofibroblasts in hypertrophic scar can provide an analogous therapeutic mechanism, making these myofibroblasts susceptible to apoptosis by ABT-263, while quiescent fibroblasts in normal skin tissues are not susceptible. We found that ABT-263 selectively induced HSFs apoptosis in a concentration dependent manner through flow cytometry analysis. Further immunoprecipitation showed that ABT-263 could reduce the binding of BCL-2/BCL-XL with BIM and subsequently release the BIM, which further caused the mitochondrial membrane potential changes and finally induced the apoptosis of hypertrophic scar myofibroblasts. Furthermore, our *in vitro* experiments showed that ABT-263 could inhibit HSFs proliferation and reduce the expression of α-SMA, collagen I, and collagen III in a concentration dependent manner. These results may partially explain the beneficial role of ABT-263 in hypertrophic scar treatment.

There are several potential advantages of using ABT-263 topically to treat hypertrophic scars. Firstly, selectively inducing apoptosis of myofibroblasts by targeting the antiapoptotic protein BCL-2/BCL-XL could avoid excessive inflammatory reactions caused by excessive cell breakdown, which may aggravate scar formation (Pan et al., 2017; Moncsek et al., 2018). Secondly, previous study has reported that ABT-263 could induce platelet apoptosis and thrombocytopenia via oral systematic administration (Mullard, 2016). In this study, local injection of ABT-263 could reduce systemic toxicity of ABT-263 and increase the local agent concentration.

## Data Availability Statement

The raw data supporting the conclusions of this article will be made available by the authors, without undue reservation.

## Ethics Statement

The studies involving human participants were reviewed and approved by the Ethics Committee of Changhai Hospital, Shanghai, China. The patients/participants provided their written informed consent to participate in this study. The animal study was reviewed and approved by the Ethics Committee of Changhai Hospital, Shanghai, China.

## Author Contributions

YZ conceived and designed the study; XY and YX consulted the literature and prepared materials; XY, YX, CZ, FS, and YZ performed the experiment and analyzed the data; XY and YZ wrote the paper; YZ, SX, and ZX revised the paper.

## Funding

This work was supported by the National Key R&D Program of China (2019YFA0110503); the Youth Incubation Plan of the Military Medical Science and Technology (20QNPY035); the National Nature Science Foundation of China (82072170, 81701905, 81772076, 81871559, and 81571897); the CAMS Innovation Fund for Medical Sciences (2019-I2M-5-076); and the Shanghai health system excellent talent training program (2017BR037).

## Conflict of Interest

The authors declare that the research was conducted in the absence of any commercial or financial relationships that could be construed as a potential conflict of interest.
